# Tenascin-C and alpha-smooth muscle actin positive cells are increased in the large airways in patients with COPD

**DOI:** 10.1186/1465-9921-12-48

**Published:** 2011-04-15

**Authors:** Magnus Löfdahl, Riitta Kaarteenaho, Elisa Lappi-Blanco, Göran Tornling, Magnus C Sköld

**Affiliations:** 1 Dept Medicine, Division of Respiratory Medicine, Karolinska Institutet, Karolinska University Hospital Solna. Stockholm Sweden; 2 Inst of Clinical Medicine, Dept of Internal Medicine/Respiratory Research Unit, Centre of Excellence in Research, University of Oulu and Oulu University Hospital, Oulu, Finland; 3 Department of Pathology, Oulu University Hospital and Institute of Diagnostics, Department of Pathology, University of Oulu, Finland

## Abstract

**Background:**

Chronic obstructive pulmonary disease (COPD) is characterized by inflammation and remodeling of the lungs. This results in alterations in extracellular matrix (ECM) and structural changes leading to airflow obstruction. We studied the expression of tenascin-C (Tn-C) and alpha smooth muscle actin (α-SMA), which act as a marker of myofibroblasts, in large airways from COPD patients. Our aim was to elucidate whether this expression correlated with smoking or with disease development.

**Methods:**

Bronchoscopy was performed on 20 COPD patients (mean age 56 years; range 39-61; FEV1/FVC < 70% and FEV1 median 53% (range 33-69) of predicted). Age and smoking matched smokers (S) without COPD (n = 13) and age matched non-smokers (NS) (n = 14) served as controls. Bronchial mucosal biopsies were analyzed by immunohistochemistry. The distribution of Tn-C expression was assessed and graded in three levels, and the number of spindle shaped cells staining positive for α-SMA were counted.

**Results:**

Biopsies from COPD patients had more (P < 0.001) Tn-C expression than the two control groups. A significantly (P < 0.05) increased number of spindle shaped cells expressing α-SMA was observed in COPD patients compared with the controls. Smokers and nonsmokers did not differ in this respect. The expression of Tn-C correlated positively (P < 0.001) to the number of α-SMA positive cells.

**Conclusions:**

We demonstrate increased expression of Tn-C and α-SMA positive cells in the large airways in COPD. This was not associated to smoking *per se*, but to the presence of airway obstruction. Our findings add new information regarding remodeling characteristics and highlight the large airways as a potential site for airways obstruction in COPD.

## Introduction

Chronic obstructive pulmonary disease (COPD) is recognized as an important cause of morbidity and mortality [1], affecting 7-14% of all adults in the western world [2,3]. Tobacco smoking is identified as the most important risk factor, and the disease is associated with an abnormal inflammatory response in the lung [4].

Inflammation in COPD has been displayed at different levels within the bronchial three and in the lung parenchyma [5-7]. This inflammatory response is chronic in nature and has been associated with an increased level of profibrotic mediators such as transforming growth factor β (TGF-β) and epidermal growth factor (EGF) [8]. The major site of the airways obstruction in COPD is located in the small airways and the obstruction *per se *has been found to be associated with structural changes in the bronchioles and in the pulmonary parenchyma [9]. Fibrosis observed in the subepithelial region in the large airways is a hallmark of asthma [10] and has been shown to correlate to disease severity [11-13]. In COPD or in chronic bronchitis, some studies have shown no alteration in reticular basement thickness [14] whereas other studies have shown a thickening of the reticular basement membrane compared to controls [11,15].

Tenascin-C (Tn-C) is an extracellular matrix glycoprotein involved in tissue remodeling. Its expression is increased in the airway wall in diseases characterized by remodeling such as asthma [16,17]. In addition, Tn-C has been shown to be increased in a number of other lung diseases associated with remodelling of extracellular matrix (ECM), such as idiopathic pulmonary fibrosis (IPF), allergic alveolitis, sarcoidosis, asbestosis, cryptogenic organizing pneumonia (COP), tuberculosis, atypical mycobacteriosis, lung cancer, mesothelioma and inflammatory myofibroblastic tumor [18-20], Tn-C has also been reported to be expressed, during fetal development of the human lung [21] but not in healthy human adult lung. In many lung diseases but also during lung development, α-smooth muscle positive cells (α-SMA), which were obviously myofibroblasts, were shown to produce most of the Tn-C mRNA [22,23]. Myofibroblasts are fibroblast-like cells that were discovered in the early 70's [24]. These cells were initially defined in ultrastructural terms, with the essential features being stress fibers, well-developed cell-to-stroma attachment sites i.e. fibronexus and intercellular intermediate and gap junctions [25]. Microscopic studies demonstrated that these cells express αalpha-SMA, fibronectin and vimentin [26]. Nowadays myofibroblasts are supposed to be the elementary factors in the pathogenesis of IPF and cancers [27]. αalpha-SMA is the most commonly used marker for a myofibroblast, although not specific, since also smooth muscle and endothelial cells express this marker [28]. Vimentin is an intermediate filament which is virtually always present in mesenchymal cell lines or neoplasms. At the present, the ubiquity of vimentin in soft tissues limits its diagnostic use in differentiating cell types and it mostly serves as a positive specimen control [29]. Vimentin is also expressed in inflammatory cells [30].

Studies on Tn-C expression from patients with COPD are sparse whereas other ECM proteins have been more extensively analyzed. Krakenberg and co-authors observed that fibronectin, collagens I, III and IV, laminin and hyaluronan were enhanced in lung tissues of COPD-patients [31]. On the other hand, a recent study by Gosselink et al revealed that fibronectin is decreased in small airways of COPD patients [32]. A previous experimental study using primary human lung fibroblasts cultured from patients with COPD and asthma showed that fluticasone propionate increased the expression of fibronectin but decreased the expression of Tn-C whereas salmeterol neither affected fibronectin or Tn-C [33]. In our own recently published study precursors of collagen I and III were shown to have variable expression profiles in large and small airways of the patients with different stages of COPD [34].

Given the chronic nature of inflammation in COPD and the importance of structural changes for lung function impairment [35], our aim was to quantify measures of remodeling in the large airways in COPD compared to smokers and nonsmokers. We therefore hypothesized that the expression of Tn-C is increased in COPD similarly to many other ECM proteins. Moreover, we wanted to analyze if the number of α-SMA positive cells, which probable represent myofibroblasts, are increased in COPD. Cell-specific expression of Tn-C and α-SMA was analyzed in whole bronchial biopsy tissue area, not only in the area of the basement membrane, and the immunohistochemical findings were correlated with the clinical data of the patients.

## Materials and methods

### Patients and control subjects

Twenty patients with COPD, aged 39-61 years (mean age 57) were recruited from the Division of Respiratory Medicine, Karolinska University Hospital Solna, Stockholm, Sweden (Table 1). All patients had a post bronchodilator FEV_1_/VC<70% and FEV_1_<70% of predicted and a smoking history of more than ten pack-years. In the COPD group, three of the patients had quit smoking. These three ex-smokers had a post bronchodilator FEV_1 _of 1.06, 2.17 and 1.65 (L), and had quit, respectively, nine years, six months and ten years prior to the study entrance. None of the patients in the COPD group had clinical history or radiological signs of any other pulmonary disease than COPD. Age-matched smokers (n = 13) without COPD and non-smokers (n = 14) served as controls. The COPD patients and the control group of smokers was matched regarding smoking history assessed as pack-years. All had a normal chest X-ray. No patient or control subject had a history suggesting allergy or asthma. All patients and controls were in a stable condition (i.e. none had a respiratory tract infection within three months prior to the study), and no participant had received oral or inhaled corticosteroids during the three months preceding the inclusion. Nine of the COPD patients used bronchodilator inhalers. Four of them had a short-acting beta-agonist inhaler, three had long-acting beta-agonist inhaler and two had a short-acting antimuscarinic inhaler. In addition, six patients used oral N-acetylcysteine.

**Table 1:** Characteristics and lung function data in COPD patients, smoking controls (S) and non-smoking controls (NS).

	COPD	S	NS
**N (n)**	20	13	14

**Males (n)**	11	6	7

**Age (years)**	56 ± 5	55 ± 7	57 ± 4

**Pack-years (years)**	34 (24-43)###	36 (27-37)†††	0

**FEV1/FVC**	0.54 (0.38-0.52) ***###	0.80 (0.76-0.81)	0.84 (0.83-0.84)

**FEV1/VC**	0.48 (0.52-0.62) ***###	0.79 (0.75-0.83)	0.77 (0.76-0.80)

**FEV1 (L)**	1.58 (1.22-1.94) ***###	2.89 (2.71-3.57)	3.32 (2.75-3.94)

**FEV_1 _(% predicted)**	53 (47-60)***###	98 (95-104)†	109 (106-121)

**FEV_1 _reversibility (%)**	14 (4-19)***###	4 (0-5)	2 (0-3)

**FEV_1 _reversibility (mL)**	190 (75-265)#	90 (0-170)	70 (8-105)

Data are shown as mean and standard deviation for age, mean and inter quartile range for pack-years, median and inter quartile range for all others. Significant difference between groups is marked with * (COPD vs HS), # (COPD vs NS) and † (HS vs NS). The considered levels of significance were P < 0.05 (*, # or †), P < 0.01 (**, ## or ††) and P < 0.001 (***, ### or †††). FEV1: Forced expiratory volume in one second, measured post bronchodilation; FVC: Forced vital capacity; VC: Vital capacity.

Each participant gave an informed consent and the study had the approval from the regional ethics committee, Karolinska University Hospital, Stockholm, Sweden, approval number: 99-319.

#### Pulmonary function test

All participants performed a dynamic spirometry in a standardized manner (Vitalograph^®^, Buckingham, UK). Both slow vital capacity and forced vital capacity was performed, before and 10 minutes after inhalation with 2 doses of 0.5 mg terbutalin (Bricanyl^® ^Turbuhaler^®^; AstraZeneca, Södertälje, Sweden), and reversibility was calculated.

### Bronchoscopy and bronchial biopsies

Bronchoscopy was performed as described previously [36]. Biopsy specimens were taken by use of pulmonary biopsy forceps with smooth edge jaws (Radial Edge^® ^Biopsy Forceps, Boston Scientific, Boston, MA). Four to six endobronchial mucosal biopsies were taken from each subject, and they were all collected from lobar or segmental carinae of the upper left lobe or the apical segment of the lower left lobe.

### Processing and immunohistochemical stainings of bronchial biopsies

All biopsies were immediately formalin-fixed and embedded in paraffin. The material was evaluated, and representative tissue blocks from each case were selected for immunohistochemistry studies. Immunohistochemical stainings were performed as described previously [20,22,23,35,37,38]. Negative controls were obtained by using non-immune serum and PBS as substitute for the primary antibodies. Information of the antibodies used is shown in Table 2.

**Table 2:** List of the antibodies, concentrations and antigen-retrieval methods used in the study.

Antibody	Source	Concentration	Antigen retrieval
α-SMA	Dako	1:1000	MW 19 min in tris-EDTA‡

Desmin	Dako	1:300	MW 19 min in tris-EDTA

Tn-C	Biohit	1:1000	MW 30 min in tris-EDTA

Vimentin	Dako	1:1500	MW 14 min in citrate†

MW = microwave heat treatment; ‡ = tris/EDTA buffer, pH 9.0; † = citrate buffer, pH 6.0

### Quantification of Tn-C and α-SMA expression

Two experienced pulmonary pathologists (RK and ELB) evaluated all biopsies. When analyzing the lung samples, both pathologists were blinded to the disease group status of the patients. 1-4 biopsy samples of each patient were analyzed, but for the statistics only one sample of each case was selected. The average area of the sections was 1-2 mm^2^, and the whole tissue section was analyzed by immunohistochemistry in each case. Immunohistochemical stainings for Tn-C and α-SMA was performed in serial sections, i.e. in consecutive sections. Staining for desmin was done in 44 of the most representative cases for phenotyping the α-SMA positive cells. In addition, vimentin was evaluated in 22 cases in which the tissue material was available.

**Figure 1:**
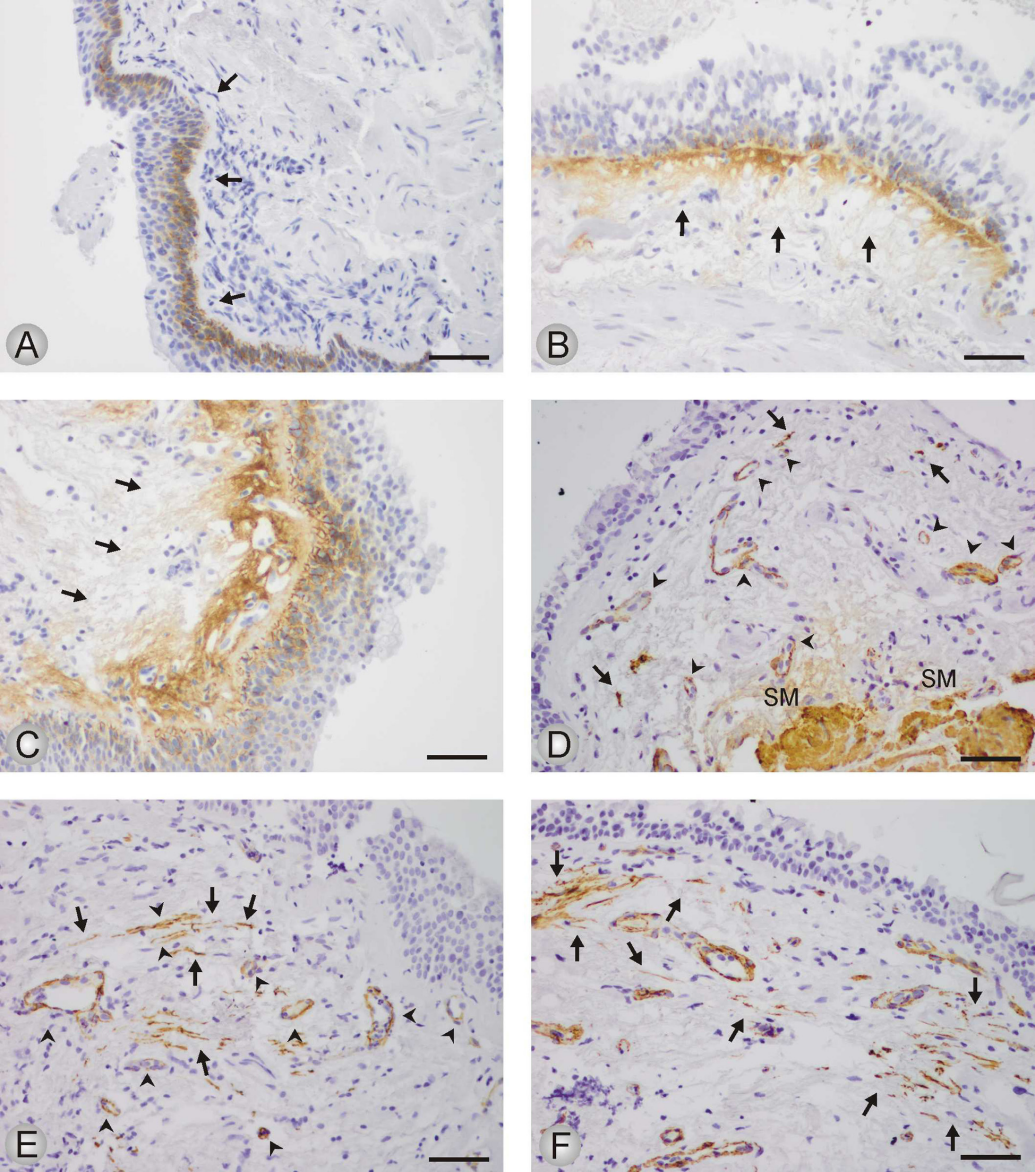
**The immunohistochemical expression of Tn-C (1 A-C) and α-SMA (1 D-F) in bronchial biopsies from study subjects**. The figures show representative microphotographs from each category. A: Positivity for Tn-C is seen in tangentially sectioned basal epithelial cells and along the basement membrane (BM) of bronchial epithelium (arrows); scale bar = 0.05 mm. B: Tn-C positivity in basal epithelial cells and in the stroma underneath the BM (arrows); scale bar = 0.05 mm. C: Positivity for Tn-C in basal epithelial cells and in a wide area of stromal connective tissue underneath the BM (arrows); scale bar = 0.05 mm. D: Spindle shaped cells positive for α-SMA (arrows) in a biopsy graded to the category 1-4 positive cells. Smooth muscle of the bronchial wall (SM) or blood vessels (arrow heads) were not counted; scale bar = 0.05 mm. E: A bronchial biopsy graded to the category 5-10 α-SMA positive cells (arrows). Blood vessels (arrow heads) or smooth muscle layer of the bronchial wall (SM) were not counted; scale bar = 0.05 mm. F: More than 10 spindle shaped cells are showing positivity for α-SMA (arrows) in a bronchial biopsy; scale bar = 0.05 mm.

The quantitative expression of Tn-C was assessed in three categories. *Tn-C (a)*: staining present in basal epithelial cells and basement membrane of the bronchial epithelium; *Tn-C (b)*: staining present as in *Tn-C (a) *and in the stroma underneath the basement membrane; *Tn-C (c)*: staining present as in *Tn-C (b) *and in the wider area of the connective tissue of bronchial walls. Representative microphotographs for Tn-C are displayed in Figure 1A-C.

The expression of α-SMA was assessed in spindle shaped cells which were obvious myofibroblasts. Smooth muscle cells and cells of vessels were not scored. Quantification of the staining was assessed in four categories. *SMA (a)*: no cells; *SMA (b)*: 1-4 cells; *SMA (c)*: 5-10; *SMA (d)*: >10 cells stained for α-SMA. See Figure 1D-F for representative microphotographs of the expression of α-SMA.

### Statistical analysis

Descriptive data on the study population were analyzed by Kruskal-Wallis ANOVA and median test for differences between the three groups and by Mann-Whitney test for comparison between two groups.

To analyse differences on immunohistochemistry between the groups, we employed a proportional odds analysis for categorical data. For Tn-C, the two odds ratios *Tn-C (a) *vs. *Tn-C (b+c) *and *Tn-C (a+b) *vs. *Tn-C (c) *were assumed to be the same within the pair wise comparison between the groups. For α-SMA expression, the three odds ratios *SMA (a) *vs. *SMA (b+c+d)*, *SMA (a+b) *vs. *SMA (c+d) *and *SMA (a+b+c) *vs. *SMA (d) *were assumed to be the same within the pair wise comparison between the groups. The proportional odds model fits data well as demonstrated by the estimates being close to the observed frequencies. Test for homogeneity, i.e. no difference between all three groups, was statistically significant for both Tn-C (p = 0.003) and α-SMA (p = 0.039), but since the difference between the HS and NS groups was small, we expressed the main effect by comparing the COPD group to HS and NS combined (geometric mean of the odds). Correlations between Tn-C and α-SMA expression and lung function were calculated with Spearman's rank correlation coefficient.

A significance level of 5% was applied for all statistical tests, and in case of a statistically significant result the probability value (p-value) is given.

## Results

### Immunohistochemistry for Tn-C

**Figure 2:**
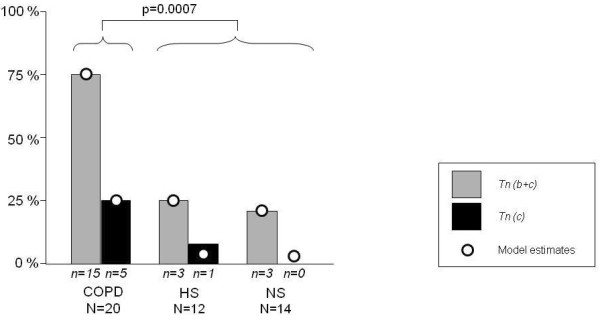
**Number and proportion of subjects expressing Tenascin C outside the basal epithelial cells and basement membrane**. Data is given for the three groups COPD, smoking controls (S) and non-smoking controls (NS). Tn C (b+c): All subjects with expression outside the basal epithelial cells and basement membrane. Tn C^©^: Subjects with expression within connective tissue beyond the stroma underneath basal membrane. The number (n) of individuals in each category is presented underneath corresponding bar. The Odds ratio for a COPD patient to be in a higher category is statistically increased (P < 0.001) compared to subjects in the control groups.

In general, Tn-C was expressed as extracellular thin and linear fibers underneath the bronchial epithelium and also in the wider area of connective tissue of the bronchial walls. All evaluated biopsies showed positivity for Tn-C also in basal epithelial cells but the expression profile varied considerable between different patients. Representative microphotographs are shown in Figure 1A-C. The number and proportion of subjects within each staining category are presented in Table 3 and Figure 2. As shown, both the numbers of patients and the proportion of subjects expressing Tn-C staining beyond basal epithelial cells and basal membrane, was higher in COPD patients compared to smokers and nonsmokers (P < 0.001). Of the three ex-smokers in the COPD group, two were in the lowest staining category *(a)*, and one in the intermediate *(b)*.

**Table 3:** The expression of Tenascin-C and α-SMA in patients with COPD, smokers (S) and non-smokers (NS)

	COPD	S	NS
**Tenascin C (Tn-C)**			

Number of acceptable biopsies	20	12	14

Subjects expressing Tn-C only in basal epithelial cells and basal membrane, *Tn-C (a)*	5 (*25%*)	9 (*75%*)	11 (*79%*)

Subjects expressing Tn-C as *Tn-C (a) *plus the stroma underneath basement membrane, *Tn-C (b)*	10 (*50%*)	2 (*17%*)	3 (*21%*)

Subjects expressing Tn-C as *Tn-C (b) *plus wider expression within connective tissue, *Tn-C (c)*	5 (*25%*)	1 (*8%*)	0 (*0%*)

**α-SMA**			

Number of acceptable biopsies	18	13	12

Subjects with no cells expressing α-SMA, *SMA (a)*	3 (*17%*)	7 (*54%*)	7 (*59%*)

Subjects with 1-5 cells expressing α-SMA, *SMA (b)*	6 (*33%*)	2 (*15%*)	3 (*25%*)

Subjects with 5-10 cells expressing α-SMA, *SMA^©^*	4 (*22%*)	3 (*23%*)	1 (*8%*)

Subjects with >10 cells expressing α-SMA, *SMA (d)*	5 (*28%*)	1 (*8%*)	1 (*8%*)

### Immunohistochemistry for α-SMA

**Figure 3:**
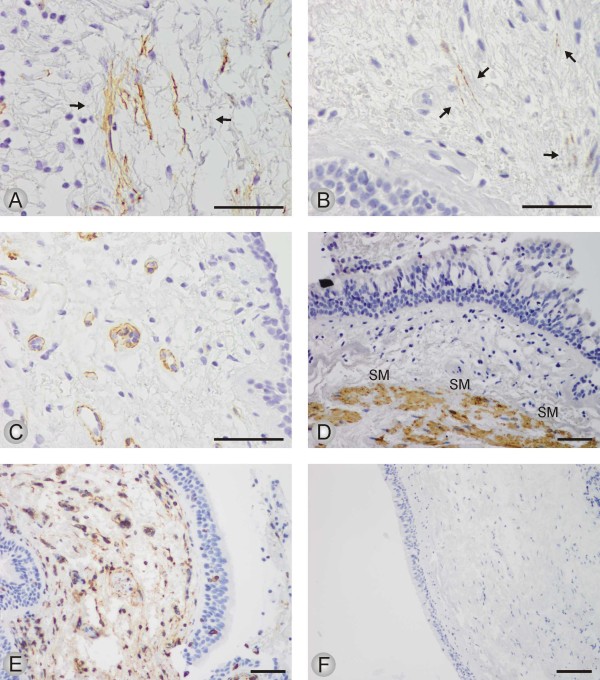
**The immunohistochemical expression of α-SMA, desmin and vimentin in bronchial biopsies from study subjects**. The figures show representative microphotographs from each category. A: High power field of spindle shaped cells positive for α-SMA; scale bar = 0.05 mm. B: High power field of desmin positive spindle shaped cells; scale bar = 0.05 mm. C: Ring like structures of blood vessels positive for α-SMA; scale bar = 0.05 mm. D: Thick bundles of smooth muscle of the bronchial wall, staining for desmin; scale bar = 0.05 mm. E: Staining for vimentin from a case in which no α-SMA or desmin positive spindle shaped cells were found. Positive staining pattern in normal fibroblasts and lymphocytes of the subepithelial connective tissue; scale bar = 0.05 mm. F: A Negative control in which the primary antibody has been substituted with non-immune mouse serum; scale bar = 0.1 mm.

Spindle shaped α-SMA positive cells were present in a proportion of subjects from all three study groups, representative microphotographs are shown in Figure 1D-F and Figure 3A. Out of the 44 cases with available stainings for desmin, 17 cases were spindle shaped cells positive both for α-SMA and desmin, and 15 cases were spindle shaped cells positive for α-SMA but negative for desmin. In the remaining 12 cases no spindle shaped cells positive for either α-SMA or desmin were found which finding indicate that those cases did not revealed any myofibroblasts. In the cases with spindle shaped cells positive for both antibodies, the desmin positive cells were always very few in numbers (Figure 3B). The α-SMA positive smooth muscle cells and endothelial cells were excluded by their different location and morphology when compared to that of spindle shaped cells (Figure 3C-D).

**Figure 4:**
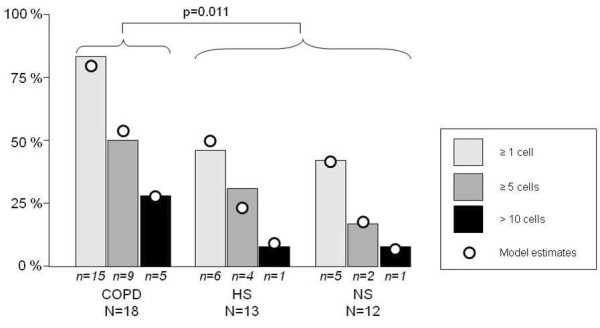
**Number and proportions of subjects with cells staining positive for α-SMA**. Data is given for the three groups COPD, smoking controls (S) and non-smoking controls (NS). The numbers (n) of individuals in each category is presented as digits underneath each bar. The odds for a COPD patient to be in a higher category is statistically increased (P < 0.05) compared to subjects in the control groups.

The number and proportion of subjects within each staining category are presented in Table 3 and Figure 4. Presence of α-SMA staining was observed in 83% of the COPD patients, in 46% of the smokers and in 41% of the nonsmokers. When data are presented as cumulative number and proportion of individuals with increasing number of cells stained positive for α-SMA, COPD patients had significantly higher (P < 0.05) α-SMA expression than smokers and nonsmokers.

Of the three ex-smokers in the COPD group, one was in category *(b)*, and two were in category *(c)*.

### Immunohistochemistry for vimentin

Regardless of the presence of α-SMA or desmin positive spindle shaped cell, all cases expressed vimentin positive slender stromal cells. Most of them were probably fibroblasts of the subepithelial connective tissue (Figure 2E). In addition to this, all inflammatory cells stained positively for vimentin.

### Correlation between Tn-C and α-SMA

**Figure 5:**
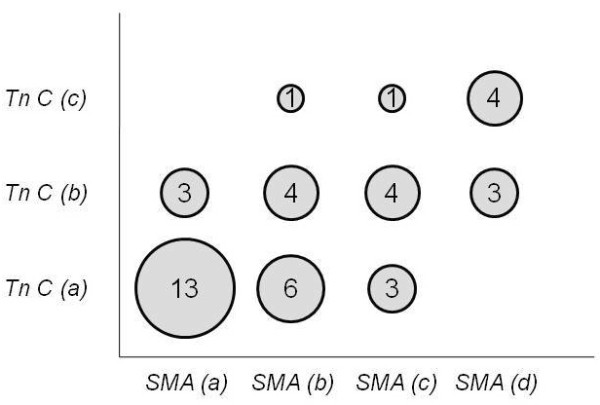
**Correlation between staining for Tenascin C and α-SMA in all subjects**. Increasing degree of staining is indicated by categories *a-c *and *a-d*. Number in the circles indicate number of subjects. The estimate for the correlation coefficient was 0.6; P < 0.0001.

The expression of Tn-C correlated positively to the expression of α-SMA. The estimate for the correlation coefficient was 0.6; P < 0.0001 (Figure 5).

### Correlations between Tn-C and α-SMA expression and lung function parameters

There was no correlation between the expression of Tn-C or α-SMA and any parameter of pulmonary function (data not shown).

## Discussion

In this study, we investigated by immunohistochemistry the expression of Tn-C and α-SMA positive spindle shaped cells in bronchial mucosal biopsies as measures of remodeling of large airways in patients with COPD. We found that COPD patients had more expression of both Tn-C and α-SMA positive cells compared to controls. In addition, there was a positive correlation between Tn-C and α-SMA expression. There were, however, no correlations between the expression of Tn-C or α-SMA and any lung function parameter.

Due to the differential immunohistochemical expression of Tn-C and α-SMA we evaluated them in two different ways: Because the expression of Tn-C was mainly extracellular and exhibited considerable variations between individual patients, its expression was analyzed by an applied semiquantitative method which took into consideration the specific cellular and histopathological localizations of the protein also in the areas around basement membranes. In contrast, α-SMA expression was mainly intracellular also in those spindle shaped cells which were quantitatively counted in the present study. Both these evaluation methods are easily applied in routine clinical diagnostics since no extra equipments is needed. For further development of our grading systems the use of computer-assisted tomography might be beneficial. Our method has not been widely used and its repeatability may be lesser than 3-dimensional or 2-dimensional methods described previously [39].

Tn-C is a glycoprotein associated with tissue remodeling. In a study by Liesker *et.al*. [15], an increase in Tn-C expression in the large airways was seen both in COPD and in asthma patients. There was, however, no difference between COPD patients and a matched ex-smokers control group. There are several differences between the study by Liesker *et.al*. and the present study. Firstly, our study has two control groups: smokers and non-smokers. Since there were no differences in Tn-C expression between smokers and non smokers in our study, we believe that the increased expression seen in the COPD group is associated with the disease, i.e. airways obstruction rather than exposure to tobacco smoke. Secondly, in our study, quantitatively more patients with a longer duration of smoking were investigated, and the COPD patients had a more severe airway obstruction. Finally, in our study all COPD patients except three were current smokers, and all subjects in the smokers control group were present smokers.

A previous study by Laitinen *et.al*.showed an increased expression of Tn-C in the subepithelial layer of the basement membrane of patients with asthma when using immunofluorescence and morphometric methods for analyzing the bronchial biopsy samples [17]. The quantification method of Tn-C in the present study was not similar to the study of Laitinen *et.al*. Since we analyzed the immunohistochemical expression of Tn-C in the specific histological localizations of the airway mucosa instead of measuring it. Furthermore, we observed that the staining for Tn-C beyond basal epithelial cells and basement membrane was higher in COPD patients compared to that of smokers and nonsmokers. The results of our study are somewhat similar to that particular study in that respect that in both studies the increase of Tn-C seemed to be correlated with the remodeling process of the airways, and not to its trigger. To our knowledge, not much attention has previously been paid on Tn-C expression outside the basement membrane area. We observed, however, that over 50% of our COPD-patients showed an increased expression of Tn-C beyond the basement membrane area. Laitinen and co-workers analyzed also the number of eosinophils and lymphocytes, but did not found any correlation between the amount of these inflammatory cells and the expression of Tn-C. In the present study we attempted to compare the number of α-SMA positive spindle shaped cells, which were obviously myofibroblasts, with the amount of Tn-C and found a positive correlation between these two markers.

We were also able to show that biopsies from every subject displayed a positive immunohistochemical expression for Tn-C at least around basal cells of the bronchial epithelium, a somewhat novel finding since Tn-C has not regularly been shown to be expressed in normal adult lung tissue. The results of our study might signify that there is some constitutional expression of Tn-C in basal epithelial cells of the human bronchus. In our previous studies in normal developing human lung the expressions of Tn-C protein and mRNA were increased during early developmental stages, and decreasing in the end of gestation [23]. In the normal adult human lung Tn-C expression was observed to be very sparse [40], although in our earlier studies we focused mainly on the alveolar level, not the central airways. However, the enhanced Tn-C expression, co-localized with the expression of myofibroblasts, has been observed in small airways i.e. bronchioles of human lung in neonatal disorders such as respiratory distress syndrome (RDS) and bronchopulmonary dysplasia (BPD) [37].

Both in pulmonary fibrosis and during lung development α-SMA positive spindle shaped cells, which were obviously myofibroblasts, seemed to be the main source of mRNA of Tn-C by in situ hybridization method [22,23,37]. Myofibroblasts were initially defined in ultrastructural terms, with the essential features of intracellular fibers, which are positive for α-SMA, which is nowadays the most common, yet not specific, marker for a myofibroblast [24,41]. The origin of myofibroblasts is still unclear. In our earlier studies human lung fibroblasts were differentiated into myofibroblasts by exposing cells to transforming growth factor beta (TGF-β). We observed that ultrastructural features of myofibroblasts were detected after exposure, e.g. α-SMA positive bundles in the cytoplasm of cells, extracellular fibronectin-containing structures on the surface of the cell, and extracellular Tn-C in the vicinity of the cell [38]. Myofibroblasts seemed to have a role of the remodelling process of airways in asthmatic lung at least in animal and experimental models [42,43], but not much is currently known about the expression profile and function of myofibroblasts in COPD. To our knowledge this is the first study showing that α-SMA positive cells, which might be myofibroblasts, are increased in the airways of the patients with COPD, and moreover, the number of myofibroblasts correlated with the amount of Tn-C. The results suggest that most α-SMA positive cells revealed typical expression profile of myofibroblast being positive for α-SMA, vimentin and negative for desmin. In the minority of cases the α-SMA positive cells were positive also for desmin, which may suggest the other known phenotype for myofibroblast [25]. Interestingly, α-SMA positive cells were not present in every patient, whereas Tn-C positivity, at least in basal epithelial cells, was observed in every patient studied, which may indicate that the basal cells might be able to produce Tn-C in large airways even in healthy lung, and that myofibroblasts may be responsible for the production of the excess of Tn-C in patients with COPD.

The clinical relevance of our finding can only be speculated. Hypothetically, increased ECM deposition in the large airways in our COPD patients may contribute to airways obstruction. It is, however believed that the major site of airways obstruction in COPD is in the small airways and increased airway wall thickness has been shown to correlate with FEV_1 _[9,44]. It is therefore likely to believe that the COPD patients in the present study also have features of remodeling in the small airways and probably also emphysema. Studies evaluating both large and small airways in a well characterized patient material should therefore be encouraged.

In conclusion, patients with COPD, but not smokers, have signs of airway remodelling in the large airways as measured as an increased expression of Tn-C and α-SMA positive cells which were obviously myofibroblasts. The finding may represent processes leading to structural changes in the airway wall causing lung function impairment in COPD.

## Conflict of Interest disclosures

The authors declare that they have no competing interests.

## Authors' contributions

ML was corresponding author, enrolled and characterized study participants, performed bronchoscopies and drafted the manuscript, RK and ELB performed all immunohistochemical analyses and evaluations, and participated in writing the manuscript, GT performed statistical analyses and participated in writing the manuscript, MS initiated the project, participated in its design and coordination, performed bronchoscopies, and participated in writing the manuscript. All authors read and approved the final manuscript.
